# AtPrx71-mediated regulation of stem elongation, gravitropic response, and IAA accumulation in *Arabidopsis*

**DOI:** 10.1007/s00425-025-04826-7

**Published:** 2025-09-29

**Authors:** Mami Kurumata-Shigeto, Zhou Ziyao, Diego Alonso Yoshikay-Benitez, Koki Fujita, Yosuke Iwamoto, Jun Shigeto, Yuji Tsutsumi

**Affiliations:** 1 Research in Collaborative Sciences Enabling the Future, Hiroshima University, 1-3-2, Kagamiyama, Higashihiroshima, 739-8511 Japan; 2 Faculty of Agriculture, Kyushu University, 744, Motooka, Nishi-Ku, Fukuoka, 819-0395 Japan

**Keywords:** *Arabidopsis thaliana*, AtPrx71, Gravitropism, IAA catabolism, Plant peroxidase, Stem growth

## Abstract

**Main conclusion:**

We demonstrated that *Arabidopsis* peroxidase AtPrx71 inhibits stem growth and gravitropism response via IAA catabolism, and speculate that other vascular plants, including poplar, may have the same functional peroxidase.

**Abstract:**

Poplar peroxidase CWPO-C, which exhibits significant substrate versatility, has been suggested to participate in IAA catabolism. We previously demonstrated that AtPrx71, which shares the highest amino acid sequence identity with CWPO-C (68%) among *Arabidopsis thaliana* peroxidases, also possesses similar substrate versatility. Building on these findings, we hypothesized that AtPrx71 may have a function similar to that of CWPO-C in *Arabidopsis*. Accordingly, we analyzed the expression of *AtPrx71* and examined whether *AtPrx71*-deficient mutant (*atprx71*) and *AtPrx71*-overexpressing transgenic *Arabidopsis* (*35S::AtPrx71*) lines exhibited altered IAA-related phenotypes. Expression analysis revealed that *AtPrx71* was strongly expressed in immature organs and tissues, including the upper part of the stem, which was generally consistent with that of *CWPO-C*. Furthermore, the sites of high expression include many organs and tissues where auxin accumulates. With respect to stem growth, IAA accumulation and gravitropic response, the phenotypes of the *atprx71* mutant and *35S::AtPrx71* lines were also consistent with the hypothesis that AtPrx71 is involved in IAA catabolism in developing stems. Finally, the amino acid sequences of CWPO-C and AtPrx71 are highly conserved among many land plants, especially dicots. Therefore, the IAA catabolic mechanisms discussed here are not restricted to poplar and *Arabidopsis*.

**Supplementary Information:**

The online version contains supplementary material available at 10.1007/s00425-025-04826-7.

## Introduction

Elucidation of indole-3-acetic acid (IAA) biosynthesis, inactivation, and degradation pathways is important for understanding the basic mechanisms used to control plant growth. To date, at least four inactivation pathways, mediated by dioxygenase for auxin oxidation 1 (DAO), IAA carboxyl methyltransferase1 (IAMT1), UDP-glucosyltransferase (UGT84B1), and Gretchen Hagen 3 (GH3), have been identified in *Arabidopsis thaliana* (Solanki and Shukla 2023). The major catabolic pathways that regulate plant IAA levels are thought to involve the irreversible oxidation of IAA to oxIAA (2-oxindole-3-acetic acid), followed by further glycosylation to oxIAA-glc. These reactions are catalyzed by DAOs and GH3, respectively (Kai et al. 2007; Kubeš et al. 2012; Novák et al. 2012; Pěnčík et al. 2013). Recent research has also shown that DAOs use IAA-amino acid conjugates—such as IAA-Glu and IAA-Asp—rather than IAA as substrates *in planta*. These conclusions are based on evidence that IAA levels are not reduced in DAO-overexpressing plants and that such plants do not show reduced enzyme activity (Hayashi et al. 2021). On the other hand, for more than 20 years some studies have reported that plant peroxidase (Prx) may possess IAA oxidase activity (Gazaryan et al. 1999), which suggests that it may regulate endogenous auxin levels. For example, one study found that CpPrx01, a zucchini Prx, regulates auxin levels via auxin oxidase activity (Cosio et al. 2009). However, the presence of similar Prx genes in plant species other than zucchini, as well as positive identification of the metabolites involved both remains to be confirmed. Thus, at present, there is little recognition that Prx is an enzyme that plays a common and important role in regulating IAA concentration levels in vascular plants.

The Prxs form a large gene family, with 93 genes present in *Poplar trichocarpa* (Ren et al. 2014) and 73 in *Arabidopsis thaliana* (Tognolli et al. 2002). The majority of these Prxs are secreted and function extracellularly. Interestingly, the various Prx isoforms show spatio-temporal selectivity, indicating that different Prxs play different roles (e.g., in lignification, stress resistance, cell elongation, etc.) (Shigeto and Tsutsumi 2016). However, at present, only limited knowledge is available to characterize how different Prxs target specific extracellular compounds and how Prx-mediated enzymatic reactions mediate overall plant growth. One Prx present in *Populus alba*, known as CWPO-C, has a unique oxidative activity. Concretely, it possesses the ability to oxidize sinapyl alcohol, a lignin monomer, facilitating polymerization; this function is not found in the better-known Prxs, HRP-C and AtPrx53 (Shigeto et al. 2014). Hence, CWPO-C has been considered a Prx that specifically catalyzes lignin polymerization. However, we recently found that poplar *CWPO-C* is strongly expressed in developing (i.e., immature) organs and tissues as well as in the xylem of the upper stem. In addition, heterologous overexpression of *CWPO-C* in *Arabidopsis* was found to reduce IAA levels in the upper 2 cm of the stem by up to 96% (Yoshikay-Benitez et al. 2022). Both the stem elongation rate and the stem gravitropic response also depend on *CWPO-C* levels, which suggests that poplar CWPO-C suppressively regulates IAA concentration during the early growth phase (Yoshikay-Benitez et al. 2022, 2023). Further genotypic analyses found that all dicotyledonous plants with available genome data possess a Prx with very high amino acid identity (> 60%) to CWPO-C. We found that AtPrx71, which has the highest amino acid identity with CWPO-C (68%) among the 73 *Arabidopsis* Prxs, also possesses the unique oxidative activity described above, just as CWPO-C dose (Shigeto et al. 2014). We, therefore, hypothesized that AtPrx71 and CWPO-C are the functional equivalent Prxs and share a role as enzymes that repressively regulate IAA concentration and gravitropic response in early plant growth. We then tested this hypothesis by performing an expression analysis of *AtPrx71* and phenotypic analysis using both knockout (KO) mutants and overexpression lines. Overall, this study provides insight into the universal nature of plant growth control mechanisms related to Prx-mediated stem growth and gravitropic response—possibly mediated by IAA catabolism—across all dicotyledonous plants.

## Materials and methods

### Plant materials and growth conditions

Wild-type *Arabidopsis thaliana* (ecotype Columbia, Col-0), a homozygous *A. thaliana* mutant containing a T-DNA insertion in the *AtPrx71* gene (SALK_091561), and transgenic *Arabidopsis* (*P*_*AtPrx71*_*::GUS* and *P35S::AtPrx71*) lines were used in this study. To grow plants, we first surface sterilized seeds via immersion in 0.5% (v/v) sodium hypochlorite for 5 min and then washed seeds three times in sterile water. All seeds were then sown in plastic Petri dishes containing Murashige and Skoog medium supplemented with 3% (w/v) sucrose and 0.3% (w/v) gellan gum adjusted to pH 5.6. Next, all plates were incubated in CLE-303 cultivation chambers (TOMY SEIKO Co., Ltd., Tokyo, Japan) set at 22 °C ± 1 °C under a 16 h light (100 μmol photons m^−2^ s^−1^):8 h dark cycle. Three-week-old seedlings were transferred to pots containing vermiculite and perlite (1:1, v/v), and were subsequently irrigated with 0.1% Hyponex (Hyponex Japan, Osaka, Japan) once every four days.

### Generation of ***P***_***AtPrx71***_***::GUS*** and ***35S::AtPrx71*** plants

All transgenic plants used in this study were created using the *Arabidopsis* Col-0 background. The method used to produce *P*_*CWPO-C*_*::GUS* and *P*_*AtPrx71*_*::GUS* was described by Yoshikay-Benitez et al. (2022). Briefly, to generate a fusion construct containing the *AtPrx71* promoter and a *GUS* marker gene (Supplementary Fig. S1a) and an *AtPrx71* overexpression construct consisting of the CaMV 35S promoter (Supplementary Fig. S1b), we first amplified the 1,962-bp 5′-upstream region of *AtPrx71* as well as the complete *AtPrx71* open reading frame by PCR. The amplified sequence was then subcloned into pBluescriptII (Qiagen) using restriction site cloning at the *Hind*III/*Bam*HI and *Bam*HI/*Sac*I restriction sites. PCR primer sequences were as follows: *P*_*AtPrx71*_ (5′-cttaagcttCCAAAACTATCCCACTAATTGC and 5′-ataggatccCTTGTGTTTAAATTTTAGGGTTTTAGAG); *AtPrx71* (5′-cgggatccATGGGTTTGGTTAGATCATTGTGC-3’ and 5′-cggagctcTTAATTAACCGCAGAGCAAACCC-3′). For the *P*_*AtPrx71*_::*GUS* fusion construct, we introduced the 1,962-bp 5′-upstream region of *AtPrx71* together with the *GUS* gene between *Hind*III and *Bam*HI and between *Bam*HI and *Sac*I, respectively, in the expression vector PBF2 (Nishiguchi et al. 2006). For the *AtPrx71* overexpression construct, the complete *AtPrx71* open reading frame was introduced between *Bam*HI and *Sac*I in the PBF2 vector. For each construct, the expression vector was then inserted into cells of *Agrobacterium tumefaciens* strain LBA4404. These were used to transform plants using the floral dip method (Clough and Bent 1998). Transgenic *Arabidopsis* were then selected on MS medium supplemented with 50 mg L^−1^ kanamycin. Specifically, seeds were collected from each kanamycin-resistant F1 plant, then after propagation, homozygous T2 plants were identified and selected according to the proportions of kanamycin-resistant plants. Homozygous T3 and T4 transgenic plants were used for further analysis.

### GUS staining

Transgenic *Arabidopsis* plants *(P*_*AtPrx71*_*::GUS*, *P*_*CWPO-C*_*::GUS*, and *P*_*DR5*_*::GUS*) were analyzed by GUS staining. Seedlings were fixed in ice-cold 90% acetone for 15 min. After rinsing with 100 mM phosphate buffer (pH 7.0), the seedlings were vacuum-infiltrated with GUS staining solution containing 100 mM NaPO₄ (pH 7.0), 0.5 mg/mL 5-bromo-4-chloro-3-indolyl-β-D-glucuronide (X-Gluc), 0.5 mM K₃[Fe(CN)_6_], and 0.5 mM K₄[Fe(CN)_6_] for 15 min. The seedlings were then incubated at 37 °C for 1 to 16 h. After incubation, the seedlings were washed with 70% ethanol and subsequently placed in an ethanol:acetic acid solution (6:1, v/v) to remove chlorophyll.

### RNA extraction and real-time PCR

We examined gene expression patterns in wild-type and transgenic plants. Total RNA from microdissected tissues was isolated using an RNAqueous-Micro-RNA Kit (Ambion, Inc., Austin, TX, USA). First-strand cDNA was then synthesized using ReverTra Ace^®^ (Toyobo Co., Osaka, Japan). cDNA was subsequently used as a template for real-time PCR. The sequences of the PCR primers used were as follows: for *AtPrx71* (5′-CCGGTCCGAACCTCAATCTC-3′ and 5′-CAGCCTGTTCCTTGAGTGAGAA-3′) and for the reference gene *UBQ10* (AT4G05320) (5′-ATCACCCTTGAAGTGGAAAGC-3′ and 5′-AAACCACGAAGACGC-3′). We estimated the copy numbers of fragments of each target gene using the protocol described by Takeuchi et al. (2018) with minor modifications regarding the tissue types used. Next, we normalized the relative quantities of target mRNA using *UBQ10* as an internal standard.

### Quantification of endogenous IAA

To quantify IAA content, we harvested the stems of 3.5-week-old plants and excised 2 cm segments from the tip. After the sample fresh weights were measured, they were frozen in liquid nitrogen until further analysis. Subsequent LC/MS/MS analyses, including sample preparation were carried out as described by Yoshikay-Benitez et al. (2022).

### Gravitropic bending analyses

A time-lapse camera, TLC200 (Brinno, Taipei, Taiwan) was used for all gravitropic experiments. Briefly, wild-type (WT) and *atprx71* plants with main stem lengths of about 6 cm (i.e., in plants that were ~ 3–4-week-old) were toppled horizontally so that they were illuminated by fluorescent light from a light source directly above. Next, a video was taken until gravity flexion was completed, with the time of horizontal tipping recorded as 0 min. The time that the main stem of the plant tilted 90° was measured by overlaying it with a photo taken at the start of the experiment. Mean values were determined to compare the rise times of different samples.

### Histochemical analysis

To prepare stem cross-sections for histochemical analysis, we first cut out 0.5 cm of the stem base from three 4, 5 and 6-week-old individuals of the Col-0 WT, *atprx71*, and *35S::AtPrx71-6* genotypes, respectively. Excised stem samples were first embedded in CRYOMATRIX and stored in liquid nitrogen, after which 25 µm cross-sections were prepared using a cryostat (Tissue-Tek Cryo3; Sakura Finetek, Tokyo, Japan). After cross-sections were tacked onto glass slides, the remaining CRYOMATRIX was removed via the addition of water. We then visualized the secondary cell wall and lignin distributions of cross-sections using toluidine blue (Mitra and Loqué 2014) and Wiesner staining (Euring et al. 2012) protocols with minor modifications. Briefly, sections were immersed in 0.2% toluidine blue for 5 min for toluidine blue staining and in 1% (w/v) phloroglucinol for 3 min for Wiesner staining. Cross-sections were immediately observed under an optical microscope (VHX-6000 optical microscope; Keyence, Osaka, Japan). The stained areas of the cross-sections were determined using the NIH ImageJ analysis software (https://imagej.net/ij/).

## Results

### *AtPrx71* shows gene expression patterns that are similar to those of *CWPO-C*

**Fig. 1:**
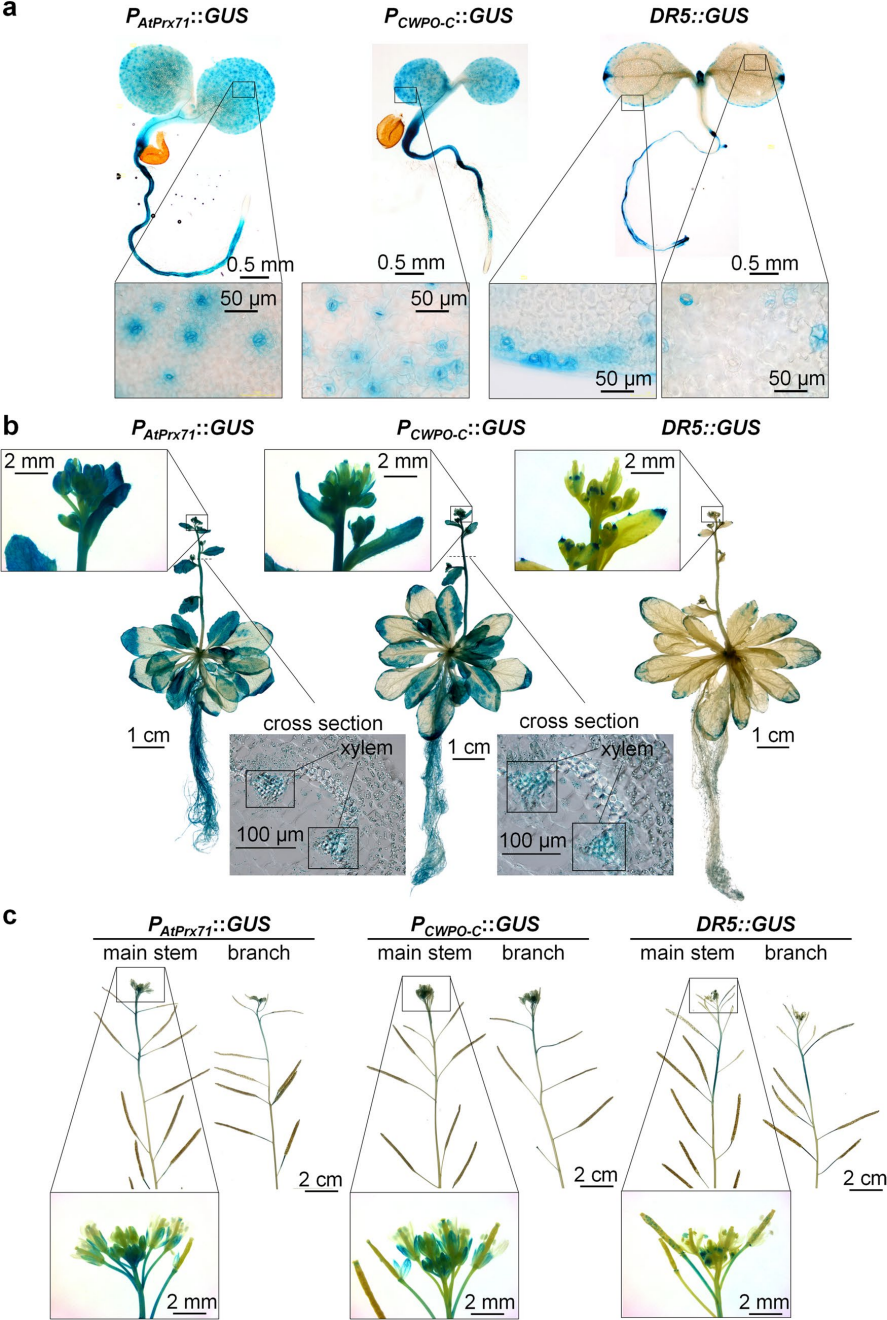
Expression of β-glucuronidase (*GUS*) driven by the promoters of *AtPrx71*, *CWPO-C*, and *DR5*. Shown are 2-day-old (**a**), 3.5-week-old seedlings (**b**) and the stems of 6-week-old plants (**c**). Two-day-old and 3.5-week-old seedlings of* P*_*AtPrx71*_*::GUS* and *P*_*CWPO-C:*_*:GUS* with reduced staining levels, as well as whole six-week-old plants, are presented in Supplementary Fig S2 and S3, respectively

The cosine similarity between the putative promoter regions of *CWPO-C* and *AtPrx71* was 0.49, indicating a moderate degree of compositional similarity in putative regulatory elements. A transformant, *P*_*AtPrx71*_*::GUS*, was generated by introducing a GUS expression cassette under the control of a 1962 bp upstream region of the translational start codon of the *AtPrx71* gene. Two days after germination, strong *GUS* expression was observed in the stomata and roots, except for the root apical meristem (Fig. 1a, Supplementary Fig. S2a). Regarding the stem, approximately 3 cm from the tip was strongly stained in 3.5-week-old plants. However, at 6 weeks of age, both the main stem and lateral branches were stained approximately 2–3 cm from the stem tip (Fig. 1b, Supplementary Fig. S2b). Cross-sections of the stained areas present on 3.5-week-old stems revealed that the xylem was particularly strongly stained (Fig. 1b, cross section). Moreover, visual inspection of the flowers of 3.5-week-old plants also showed staining of the sepals (Fig. 1b, flower), and the flowers of 6-week-old plants revealed staining on the style—i.e., in an area between the stigma and ovary (Fig. 1c, Supplementary Fig. S3). These stained sites coincided with those of the *P*_*CWPO-C*_*::GUS* transformant, which contained a *GUS* expression cassette coupled with the promoter sequence 1855 bp upstream of *CWPO-C* introduced into Col-0 plants (Fig. 1,* P*_*CWPO-C*_*::GUS*). The accumulation of auxin in plants was visualized using *DR5::GUS*, in which an expression cassette carrying the GUS gene under the control of the auxin-responsive promoter *DR5* was introduced. GUS staining was observed two days after germination in the shoot apical meristem, in peripheral leaf regions centered around hydathodes and in some stomata (Fig. 1a), as well as in the root, including the root apical meristem. In 3.5-week-old plants, staining levels in the stem were not high, whereas staining was observed in the peripheral regions of leaves and in organs thought to be anthers (Fig. 1b). In 6-week-old plants, strong staining was observed in the upper part of the stem, particularly around 4–5 cm from the tip (Fig. 1c).

### Inhibition of stem elongation by AtPrx71

**Fig. 2:**
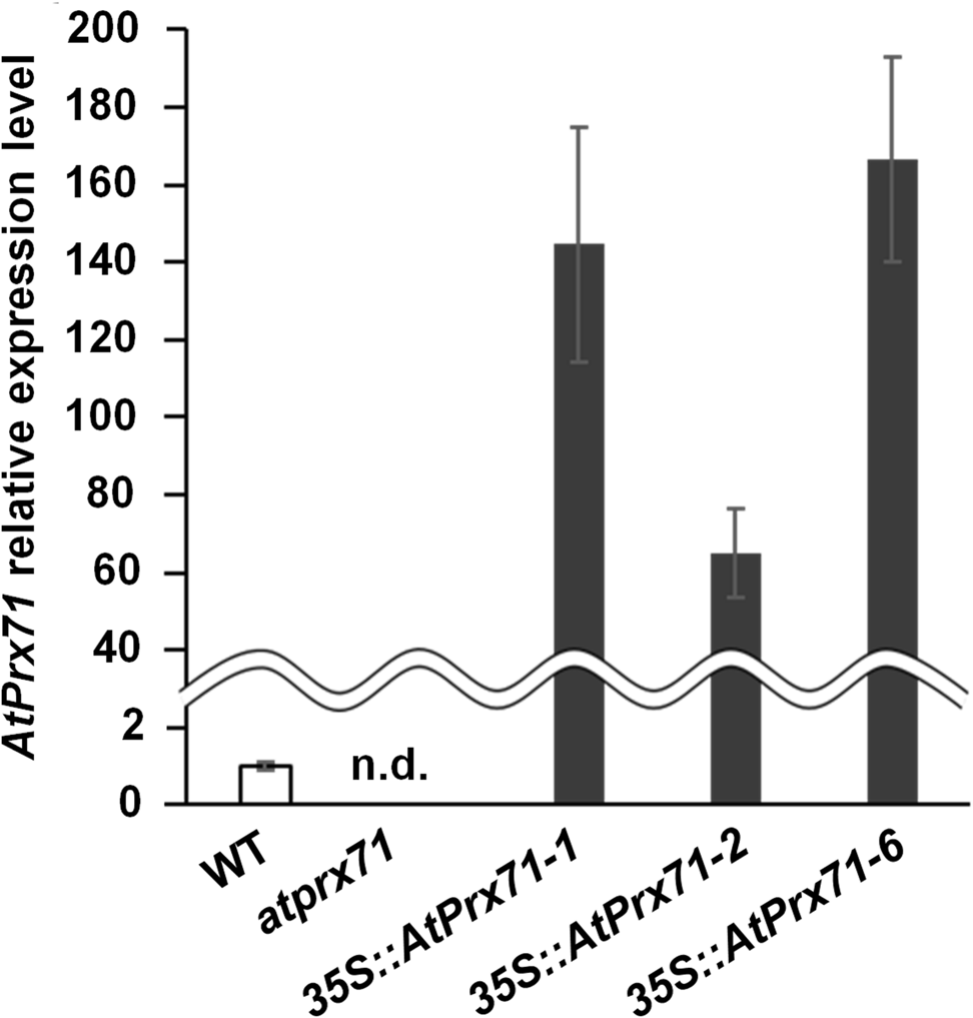
Expression of *AtPrx71* in *atprx71* and *35S::AtPrx71-1, 2, 6*. mRNA expression levels were normalized to UBQ10 (AT4G05320). Each value represents the mean ± standard deviation of three technical replicates. n.d., not detected

**Fig. 3:**
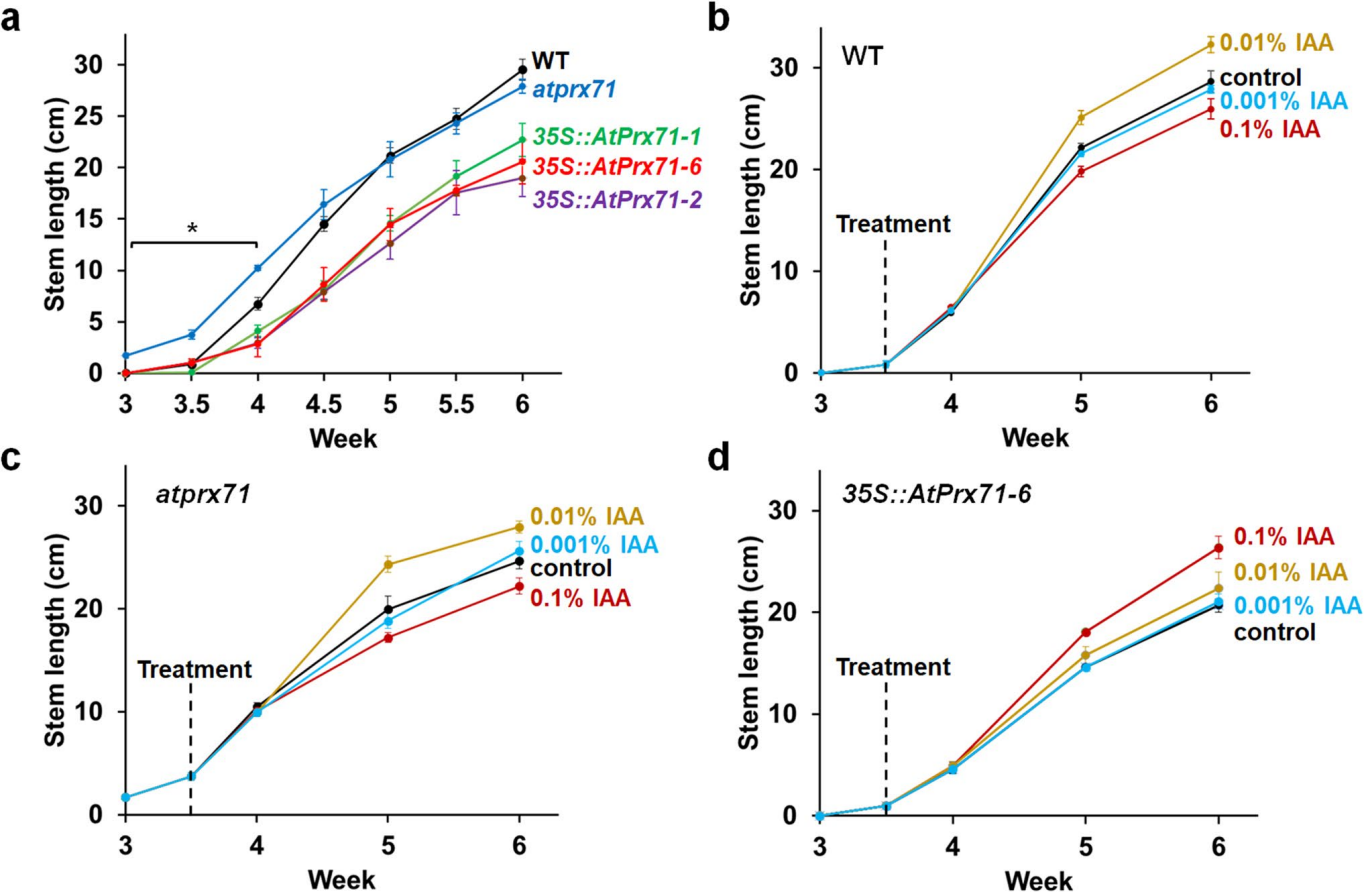
Stem elongation in wild-type, *atprx71* and *35S::AtPrx71-6* plants in response to external IAA. **a** Time course of stem length in 3- and 6-week-old wild-type (Col-0), *atprx71* plants and *35S::AtPrx71-6*. **b**–**d** Time course of stem length when 0.01% IAA was applied to the stem tip of wild-type (**b**), *atprx71* (**c**) and *35S::AtPrx71-6* (**d**). Plants treated with anhydrous lanolin, used as the solvent for IAA, served as controls. Data represent mean ± standard deviation (*n* = 5). Asterisks indicate statistical significance relative to the control (*P < 0.01, Student’s t test)

Since *AtPrx71* is strongly expressed in immature tissues and organs, we expected to observe differences in initial growth patterns between the *Arabidopsis* WT, transgenic *Arabidopsis* overexpressing the *AtPrx71* gene (*35S::AtPrx71-1*, *2*, *6*), and an *AtPrx71* knockout mutant line (*atprx71*). Figure 2 shows the relative expression levels of *AtPrx71* in WT and *35S::AtPrx71-1*, 2, 6. Here, we found no visually discernible differences in rosettes between these *Arabidopsis* lines, but we observed subsequent differences in stem elongation rates. For example, stem elongation of *atprx71* occurred approximately 2 or 3 days earlier than in the WT and resulted in a significantly longer main stem length (*i.e.,* up to 3.5 cm longer) until 4.5 weeks of age (Fig. 3). However, this difference disappeared after five weeks of age. Stem elongation inhibition was observed in *35S::AtPrx71-1*, *2*, and *6*, which showed stem lengths at 6 weeks that were 64–74% of the WT.

### Sensitivity of stem elongation to external IAA in *atprx71* and *35S::AtPrx71*

To determine whether *AtPrx71* expression was correlated with IAA sensitivity, we applied solutions containing 0.001, 0.01 or 0.1% IAA dissolved in dehydrated lanolin to the stem tips of 4-week-old WT, *atprx71*, and *35S::AtPrx71-6* plants, then measured stem length once per week. As a response, we observed differences in stem elongation between the 0.1 and 0.01% applications among lines. In the WT, we observed a 13–14% increase in stem elongation in response to 0.01% IAA after five weeks (i.e., one week after application) and six weeks (i.e., two weeks after application) relative to the negative control, which received only the dehydrated lanolin vehicle. Moreover, the application of 0.1% IAA inhibited stem growth by approximately 10% (Fig. 3b), and similar growth promotion and inhibition effects were observed in *atprx71* (Fig. 3c). In *35S::AtPrx71-6*, 0.01% IAA application exerted no effect on stem length, but 0.1% IAA application promoted stem elongation by 13–22% (Fig. 3d). One possible explanation is that, in WT and *atprx71*, the application of 0.1% IAA led to an accumulation of IAA in the stem to such levels sufficient to inhibit elongation (toxic levels), whereas in *35S::AtPrx71-6*, the excess *AtPrx71* may have degraded the applied IAA, preventing it from reaching toxic concentrations.

### IAA accumulation in *atprx71* and *35S::AtPrx71*

**Fig. 4:**
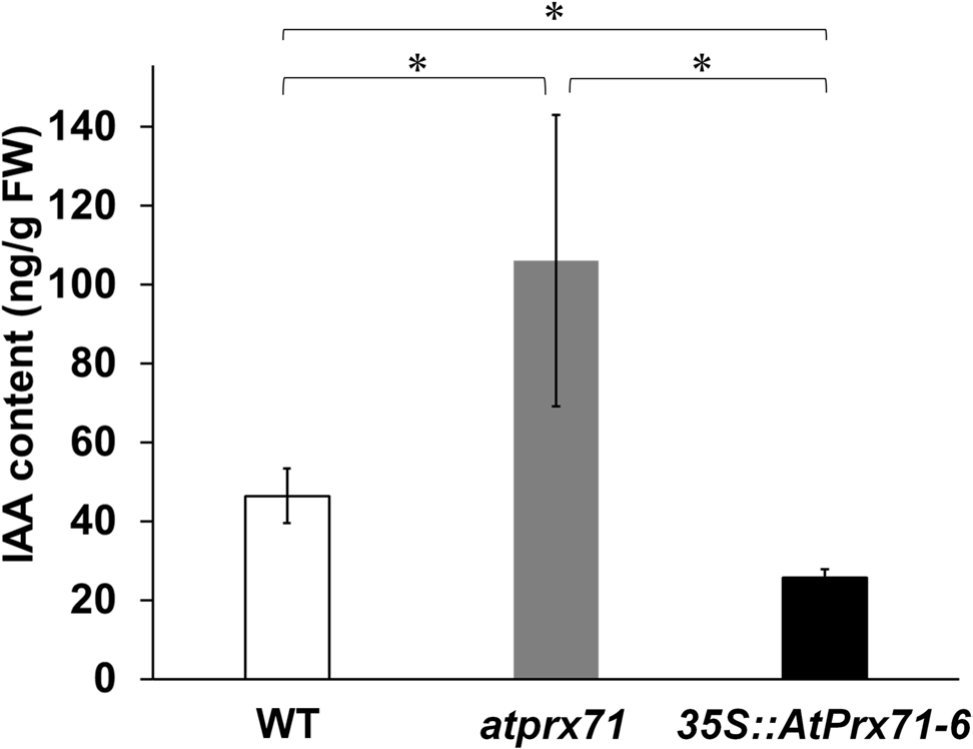
IAA content in the stem tips of five-week-old wild-type, *atprx71* plants and *35S::AtPrx71-6*. Data indicate mean ± standard deviation (*n* = 3). Asterisks indicate statistical significance relative to the WT control (*P < 0.01, Student’s t test)

If AtPrx71 directly utilizes IAA as a substrate in planta, it is expected that IAA would accumulate to higher levels in *atprx71* compared to the WT, while it would decrease in *35S::AtPrx71*. Conversely, if AtPrx71 is not involved in IAA catabolism or if—like DAO—AtPrx71 uses metabolites of IAA as substrates, the amount of IAA within mutant plants may remain unchanged. Stem samples of the 2 cm region below the tips of four-week-old WT and *atprx71* plants were therefore collected to test this hypothesis. Five samples were taken from 20 individuals, and the IAA content of each was determined. As a result, endogenous IAA levels in *atprx71* were approximately 2.3-fold higher (106 ng g⁻^1^ FW) than in the WT (46 ng g⁻^1^ FW; Fig. 4), whereas in *35S::AtPrx71*, the IAA content was approximately 55% of the WT level (26 ng g⁻^1^ FW; Fig. 4). This result strongly suggests that AtPrx71 is involved in IAA catabolism and functions directly using IAA as a substrate.

### Sensitivity of stem gravitropism in *atprx71* and *35S::AtPrx71*

**Fig. 5:**
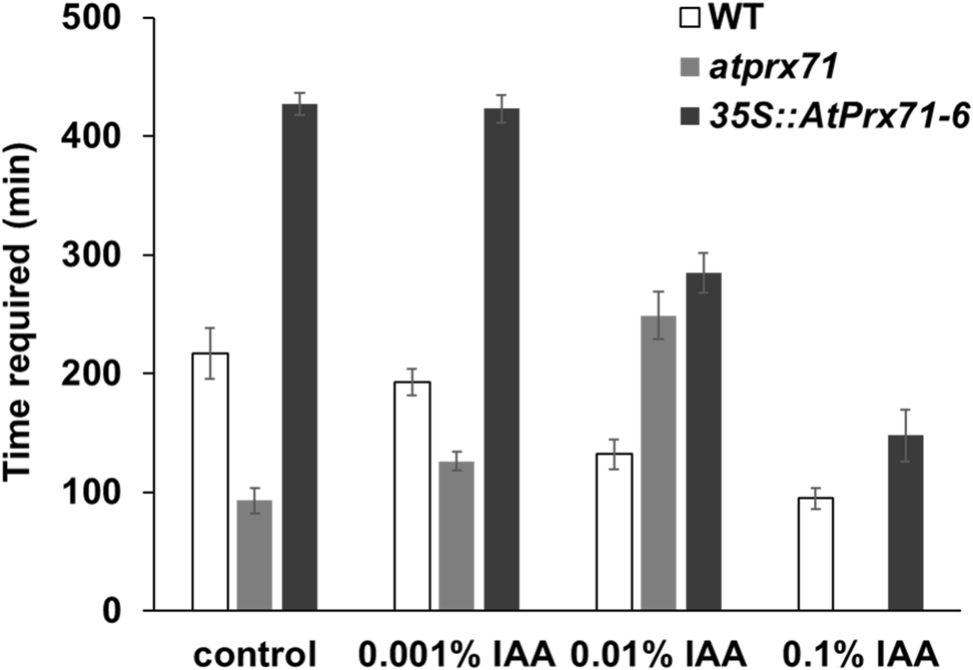
Stem gravitropic response of wild-type, *atprx71* and *35S::AtPrx71-6* plants to external IAA. Plants treated with anhydrous lanolin (used as a solvent for IAA) applied to the stem tip were used as an experimental control. Wild-type, *atprx71* and *35S::AtPrx71-6* plants (3 to 3.5-week-old, with main stem lengths of approximately 6 cm) were laid horizontally, and the time required for the stems to bend 90° was recorded

Since we observed differences in internal IAA levels within the stem as well as differences in sensitivity to external IAA among WT, *atprx71*, and *35S::AtPrx71* plants, we also expected that these plants may show differences in gravitropic response, which depends on auxin distribution. We dissolved 0.001, 0.01, 0.05 or 0.1% w/w IAA in dehydrated lanolin and applied it to the tips of stems that were approximately 6 cm in length (approximately 3–4 week-old) in representative WT, *atprx71*, and *35S::AtPrx71-6* plants. The plants were then turned over onto their sides, and we measured the time required for the stem tips to become vertical. These results are shown in Fig. 5. In the control (i.e., anhydrous lanolin vehicle only), the time required for stem erection was 217, 93 and 427 min for the WT, *atprx71*, and *35S::atprx71-6* lines, respectively. In other words, stem bending proceeded quickest for *AtPrx71*, which has a high IAA content, and was much slower in *35S::AtPrx71-6*, which has low IAA content. In both WT and *35S::AtPrx71-6*, we observed increases in stem bending in response to increasing IAA concentration. However, in *atprx71*, stem bending decreased as the IAA concentration increased, with application of 0.1% IAA completely abolishing the gravitropic response. Previous studies have reported that the addition of IAA to sunflower hypocotyls inhibited the gravity response, with even the bending response also disappearing above a certain concentration (Rorabaugh and Salisbury 1989). It is possible that when 0.1% IAA was applied to *atprx71*—which already has low IAA catabolism ability—the threshold IAA concentration at which a gravitropic response occurs was exceeded. However, WT and *35S::AtPrx71* plants have the ability to catabolize IAA and can respond to finer control over IAA production.

### Effect of *AtPrx71* expression on cell wall formation

**Fig. 6:**
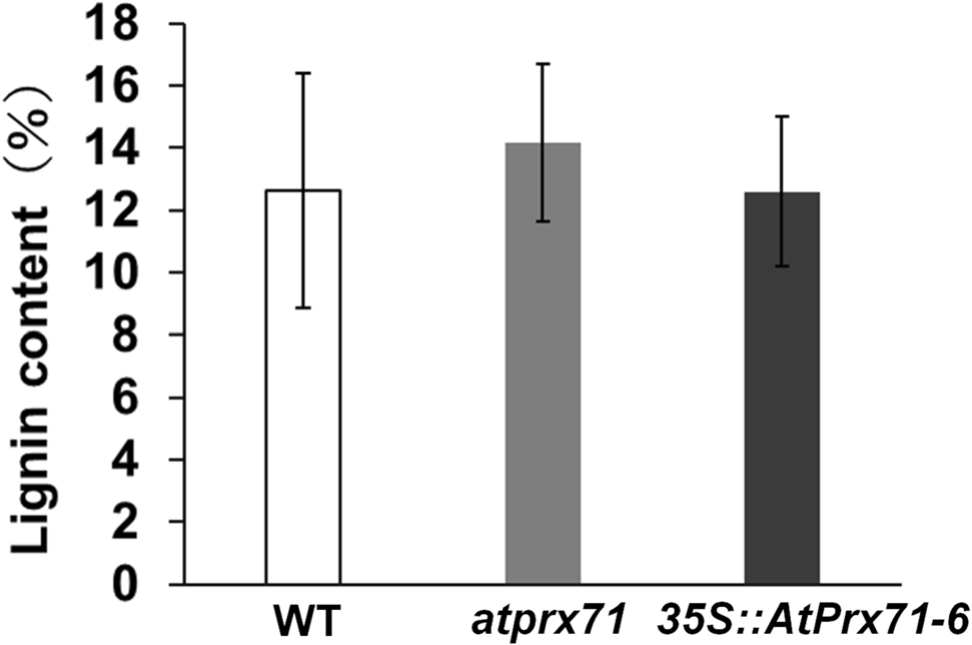
Stem lignin content in 6-week-old wild-type, *atprx71*, and *35S::AtPrx71-6* plants. Lignin content is presented as a percentage of cell wall dry weight. Data represent the mean ± standard deviation (*n* = 3)

**Fig. 7:**
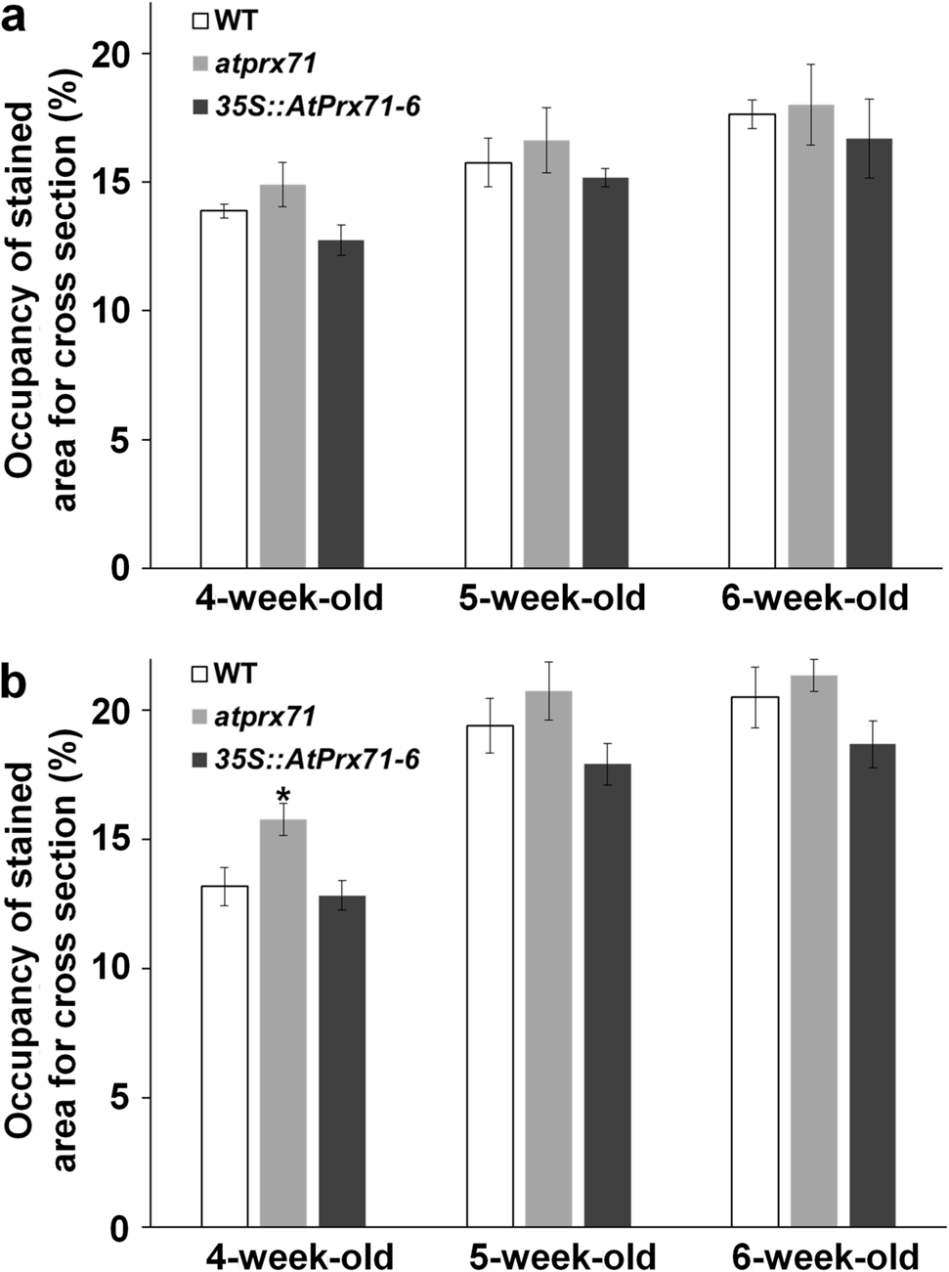
Percentage of area stained by toluidine blue (**a**) and phloroglucinol HCl (Wiesner staining) (**b**) in basal stem cross-sections sourced from wild-type (Col-0), *atprx71*, and *35S::AtPrx71-6* plants. Quantitative analysis of each staining was performed using NIH ImageJ analysis of the size of stained cross-sections. Data represent mean ± standard deviation (*n* = 3). A representative stained cross-section is presented in Supplementary Fig S4

As mentioned in the Introduction, both AtPrx71 and CWPO-C may be responsible for lignin polymerization. Since AtPrx71 is involved in cell wall formation via some polymerization of some substrates, such as lignin monomers, differences may occur in cell wall formation in each line. Therefore, we measured the lignin content of all stems and performed a histological assay of the resulting cross-sections. We found no significant differences in stem lignin content among the 6-week-old WT, *atprx71*, and *35S::AtPrx71* plants (Fig. 6). Moreover, *atprx71* did not show the phenotypes expected in response to a deficiency of cell wall component biosynthetic enzymes. This means a reduced percentage of areas stained by toluidine blue, indicating cell wall mass, and reduced areas stained by phloroglucinol staining, indicating lignin distribution (Fig. 7, Supplementary Fig. S4). The increased area stained by phloroglucinol HCl (Wiesner staining) in 4-week-old *atprx71* stems indicates that the accelerated stem elongation observed in *atprx71* from around the age of four weeks is also accompanied by progressive lignification.

## Discussion

The results obtained in this study show that the expression pattern of *AtPrx71* (Fig. 1), the suppression of stem elongation by *AtPrx71* (Fig. 3), the effect on IAA sensitivity according to the expression level of *AtPrx71* (Fig. 3 and 5), and the increase in stem IAA content in *atprx71* mutant as well as the decrease in IAA content in *AtPrx71*-overexpressing plants (Fig. 4), do not contradict the hypothesis that “AtPrx71 and poplar CWPO-C are identical functional Prx, and they suppress IAA production during early plant growth and gravitropic response analysis.” In particular, we observed how the expression levels of *AtPrx71* led to drastic changes in IAA sensitivity and IAA content in stems. Therefore, the IAA catabolic mechanism mediated by AtPrx71 may not be a minor pathway that functions in an auxiliary or support role to known IAA catabolic pathways, but may have a unique and important role in regulating the initial growth and gravitropic response of stems by regulating the amount of IAA. To fully elucidate the mechanistic basis of Prx-mediated IAA catabolism, it is necessary to identify in vivo metabolites and characterize their biological activities. Moreover, it is important to consider cooperating factors, including the hydrogen peroxide supply process, and to clarify differences in spatiotemporal roles compared with other IAA catabolic enzymes.

Previous studies have shown that AtPrx71 contributes to cell wall strengthening and the suppression of cell proliferation in *Arabidopsis* rosettes (Raggi et al. 2015). *AtPrx71* is highly expressed in immature leaves and especially in the stomata, and its effects are linked to various organs such as hydathodes, the xylem of the upper stem, and the production of immature flowers, pods, and seeds (Fig. 1). Since stomata, hydropic tissue, and the xylem are typical water-transporting tissues, it is possible that AtPrx71 plays a role in adding hydrophobicity via oxidative polymerization of substrates such as phenols, including lignin monomers, in water-transporting tissues. However, a subsequent phenotypic analysis did not find significant correlations between the amount of *AtPrx71* and the amount of cell wall or lignin molecules to be supplied to mature inflorescence stems (Fig. 6 and 7). Moreover, AtPrx71 localizes the margins of the cell, e.g., cell corners in unlignified cell wall regions within the stem (Hoffmann et al. 2020). These findings suggest that AtPrx71 may not be an essential enzyme for cell wall formation but may play roles other than lignification. For example, in *Arabidopsis*, there are as many as 73 isoforms of Prx; therefore, it is unlikely that a single enzyme is responsible for the polymerization of phenols. We also note that if *AtPrx71* is knocked out, other enzymes present can complement it. In addition, sites of significant *AtPrx71* and *CWPO*-*C* expression coincide with sites of IAA accumulation (i.e., stomata, hydathodes, etc.) (Fig. 1a), a finding that is interesting considering the substrate versatility of AtPrx71 and CWPO-C (Shigeto and Tsutsumi 2016). This is also true of the cell wall-relaxing effect of IAA, which makes it accordingly difficult to clarify the role of AtPrx71 within the cell wall control process. In other words, AtPrx71 probably regulates cell wall strength, but by one of two possible regulatory processes. The first involves regulation by synthesis of cell wall components via polymerization of polyphenol substrates, the second involves preventing cell wall relaxation through IAA catabolism-mediated regulation of IAA levels (inactivation by catabolism), and at present, it may not be feasible to completely distinguish between these regulation processes.

Since AtPrx71 shows the highest amino acid identity with CWPO-C among the *Arabidopsis* Prxs, amino acid identity may be useful for screening candidates for enzymes with the same function. The results of our comprehensive investigation of this point are shown in Suppl. Table S1. All eudicots contain at least one Prx that has an extremely high amino acid identity (> 65%) with CWPO-C. For example, in *Lotus japonicus*, Lj6g001953 and Lj6g0021815, both of which share 73% amino acid identity, and tomato Solyc01g105070, which has an amino acid identity of 77%, are potential candidates for enzymes with functions similar to those of CWPO-C and AtPrx71. In contrast, Prx sequences found in monocots show lower amino acid identities with CWPO-C or AtPrx71. For example, rice Os01g07770, which has 61% amino acid identity with CWPO-C and 57% with AtPrx71, and sorghum Sobic003G050300, which has 61% amino acid identity with CWPO-C and 56% with AtPrx71, are two of the Prxs from monocots that show the highest amino acid identity to CWPO-C and AtPrx71. An interesting exception is *Zea mays,* which shows amino acid identities to CWPO-C and AtPrx71 of 49% and 50% (for Zm00001d035336), respectively. Whether Zm00001d035336 is a Prx that has the same function as AtPrx71 and CWPO-C cannot be inferred from its amino acid sequence. There is another Prx in ferns that shows approximately 60% identity, but none were found in mosses. It is possible that most dicotyledonous plants possess Prx genes that exert the same function as AtPrx71 and CWPO-C; moreover, the IAA catabolic pathway mediated by Prx may be near-universal in vascular plants. We anticipate that the function and oxidation activity of the abovementioned Prx proteins will be clarified by future studies.

## Supplementary Information

Below is the link to the electronic supplementary material.

Supplementary file1 (DOCX 3354 KB)

## Data Availability

All data generated or analyzed during this study are included in this published article [and in its supplementary information file].

## Abbreviations

CWPO-C: Cationic cell-wall-bound peroxidase
Prx: Plant peroxidase
