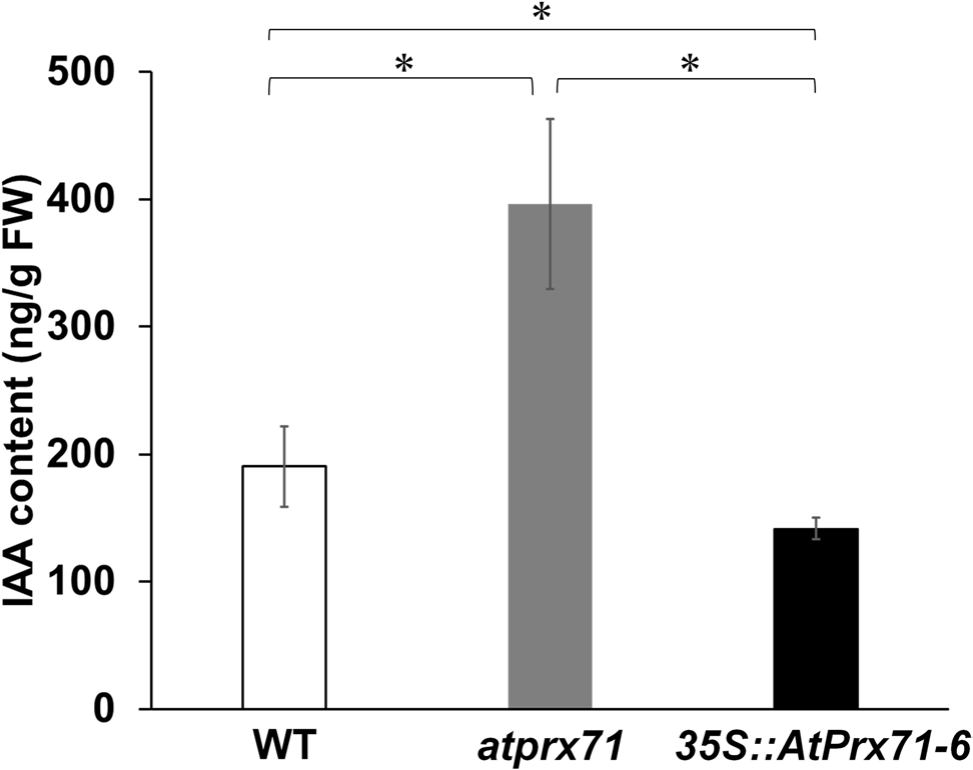# Correction: AtPrx71‑mediated regulation of stem elongation, gravitropic response, and IAA accumulation in *Arabidopsis*

**DOI:** 10.1007/s00425-025-04867-y

**Published:** 2025-11-11

**Authors:** Mami Kurumata-Shigeto, Zhou Ziyao, Diego Alonso Yoshikay-Benitez, Koki Fujita, Yosuke Iwamoto, Jun Shigeto, Yuji Tsutsumi

**Affiliations:** 1 Research in Collaborative Sciences Enabling the Future, Hiroshima University, 1-3-2, Kagamiyama, Higashihiroshima, 739-8511 Japan; 2 Faculty of Agriculture, Kyushu University, 744, Motooka, Nishi-Ku, Fukuoka, 819-0395 Japan

**Correction: Planta (2025) 262:108** 10.1007/s00425-025-04826-7

In the sentence beginning “As a result, endogenous IAA levels… in this article, numerical values are miscalculated and the correct text should read as below.

As a result, endogenous IAA levels in atprx71 were approximately 2.1-fold higher (396 ng g⁻^1^ FW) than in the WT (190 ng g⁻^1^ FW; Fig. 4), whereas in 35S::AtPrx71, the IAA content was approximately 74% of the WT level (141 ng g⁻^1^ FW; Fig. 4). This result strongly suggests that AtPrx71 is involved in IAA catabolism and functions directly using IAA as a substrate.

In this article, Fig. 4 appeared incorrectly and the corrected version is given below.