# Supplementary material for: AtPrx71-mediated regulation of stem elongation, gravitropic response, and IAA accumulation in Arabidopsis
Source: Planta. 2025 Sep 29;262(5):108. doi: 10.1007/s00425-025-04826-7 (PMC12479615; doi:10.1007/s00425-025-04826-7)
Supplement: Supplementary file 1 — Supplementary file1 (DOCX 3354 KB) [file 425_2025_4826_MOESM1_ESM.docx]

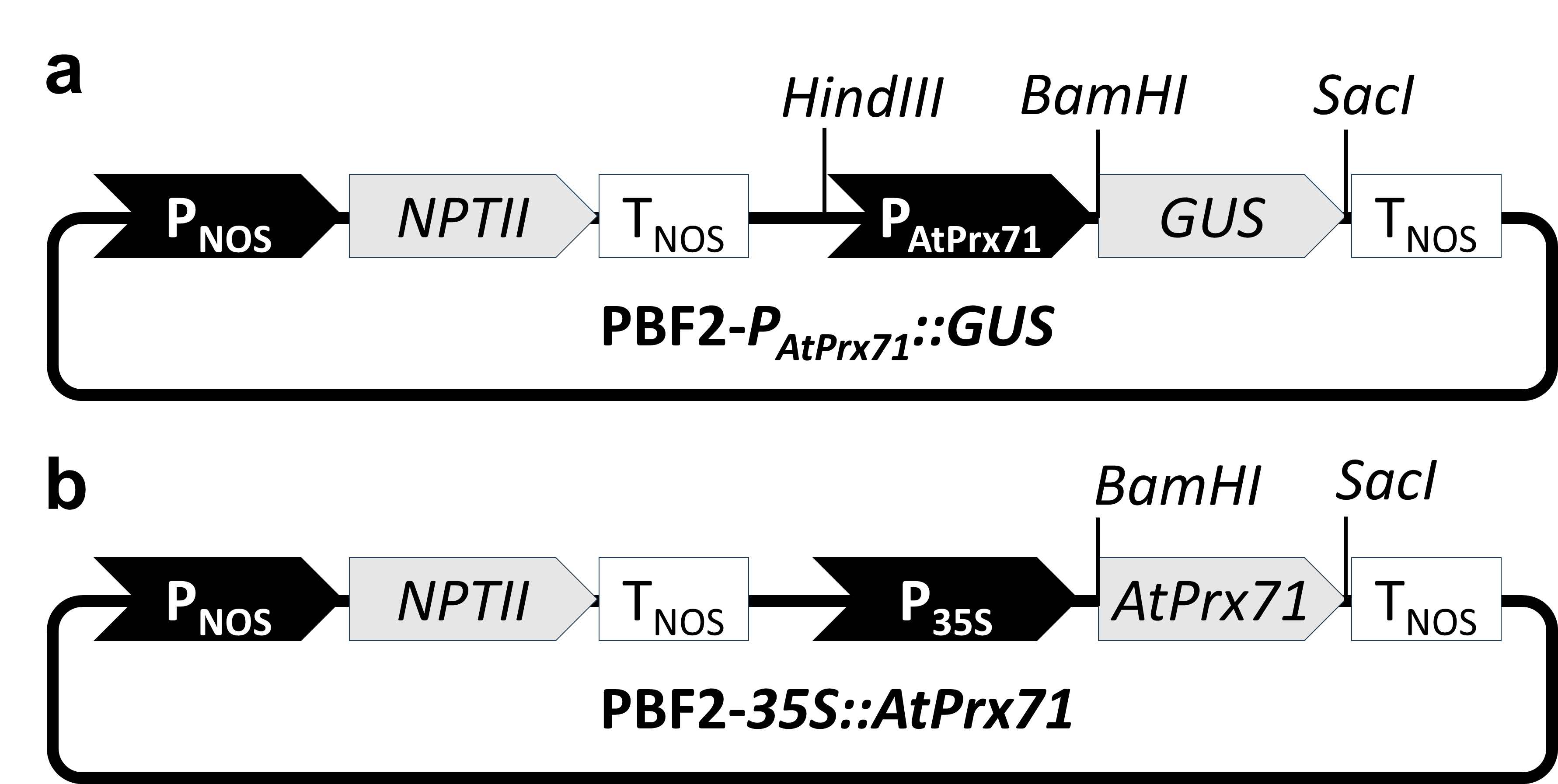


**Supplementary Figure S1** Schematic representation of an expression vector for PBF2-*P_AtPrx71_::GUS* (**a**) and PBF2-*35S::AtPrx71* (**b**). Both constructs were produced by modifying the PBF2 binary vector. P_NOS_, nopaline synthase promoter; *NPTII*, neomycin phosphotransferase gene; T_NOS_, nopaline synthase terminator; P_AtPrx71_: 1,962-bp 5′upstream region of AtPrx71gene; *GUS*, β-glucuronidase gene coding region; *AtPrx71*, cDNA of *AtPrx71*; P_35S_, cauliflower mosaic virus 35S promoter


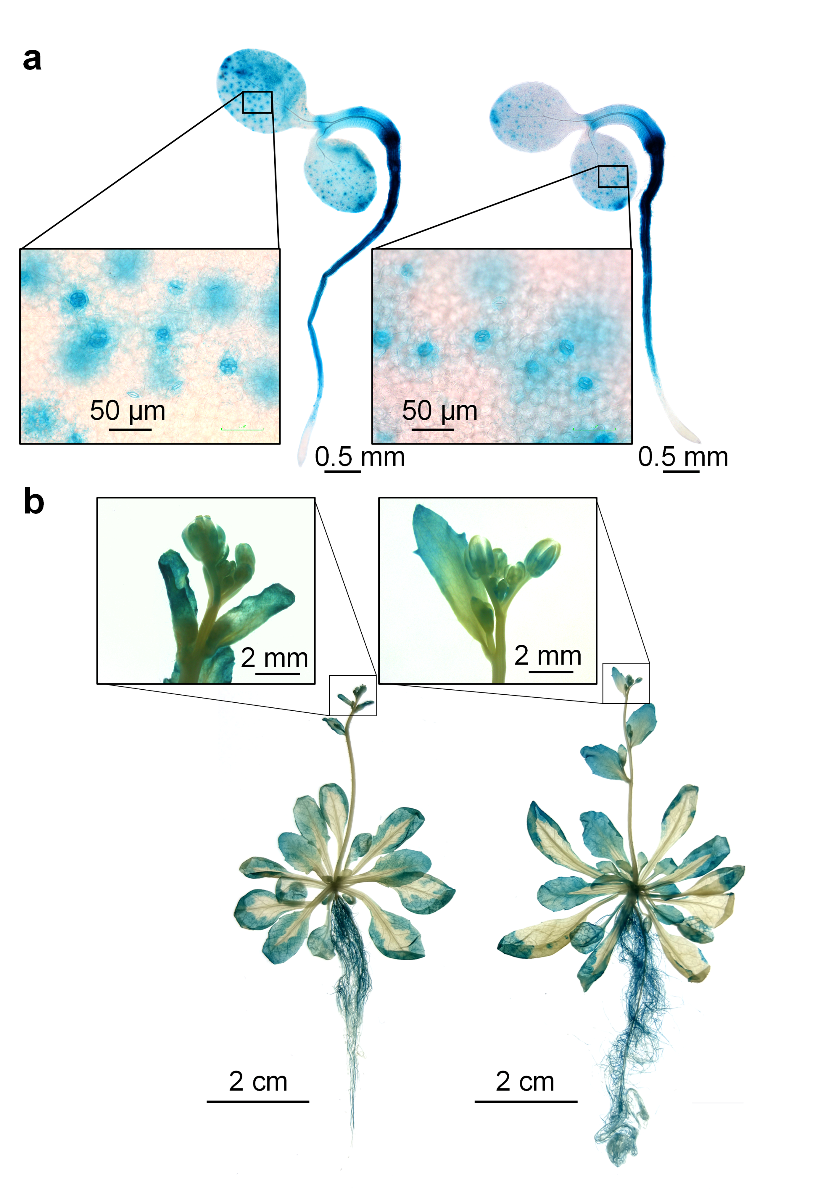


**Supplementary Figure S2** Seedlings of two-day-old (**a**) and 3.5-week-old seedlings (**b**) of *P_AtPrx71_::GUS* and *P_CWPO-C:_:GUS* with reduced staining levels. Two-day-old and 3.5-week-old *P_AtPrx71_::GUS* seedlings were incubated in GUS staining solution containing 0.5 mM X-Gul (5-bromo-4-chloro-3-indolyl-β-D-glucuronide cyclohexylammonium) at 37°C for 1 h and 2 h, respectively. Similarly, two-day-old and 3.5-week-old *P_CWPO-C_::GUS* seedlings were incubated in GUS staining solution for 1.5 h and 3 h, respectively.

**
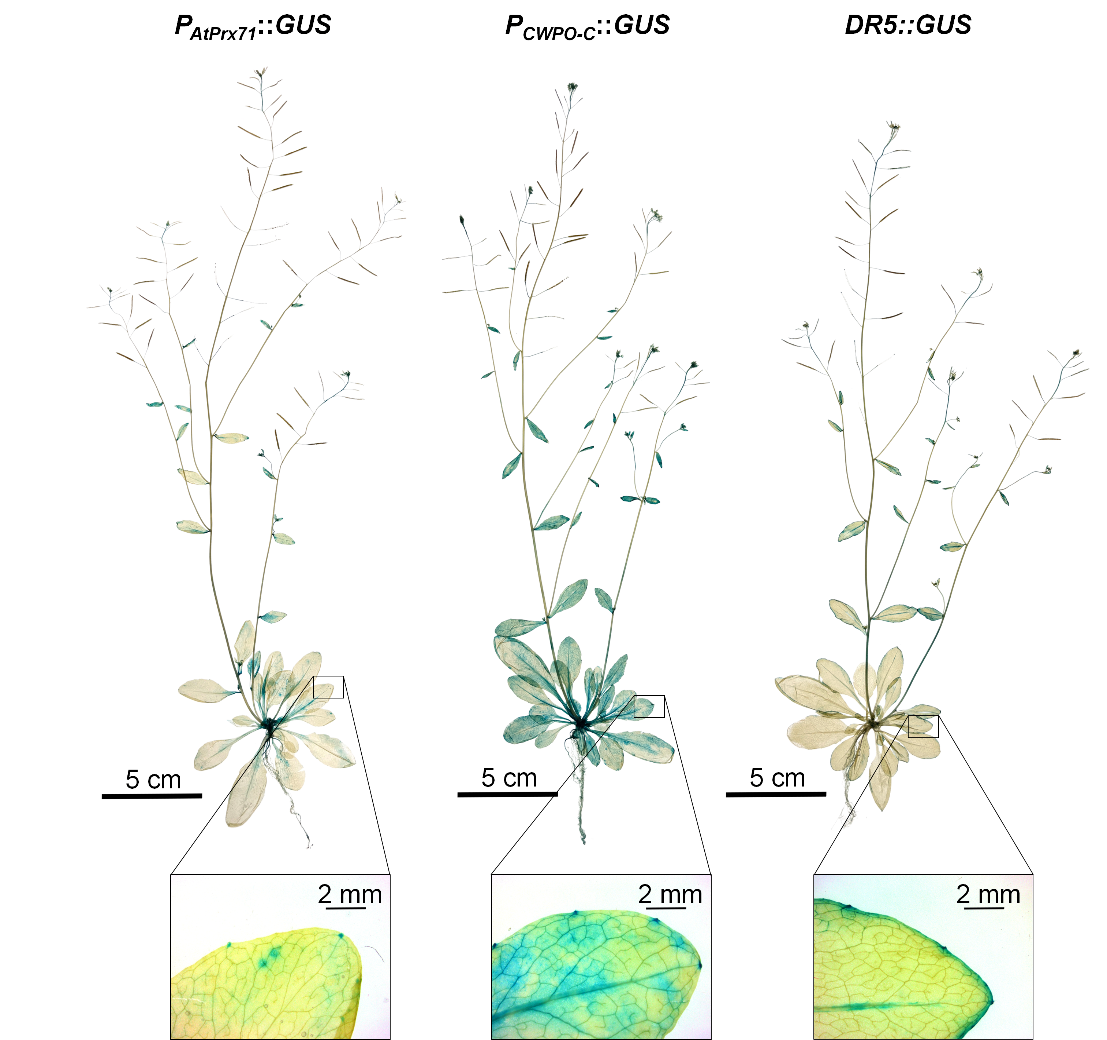
**

**Supplementary Figure S3** β-glucuronidase (GUS) expression driven by the promoters of *AtPrx71*, *CWPO-C*, and *DR5* in six-week-old plants


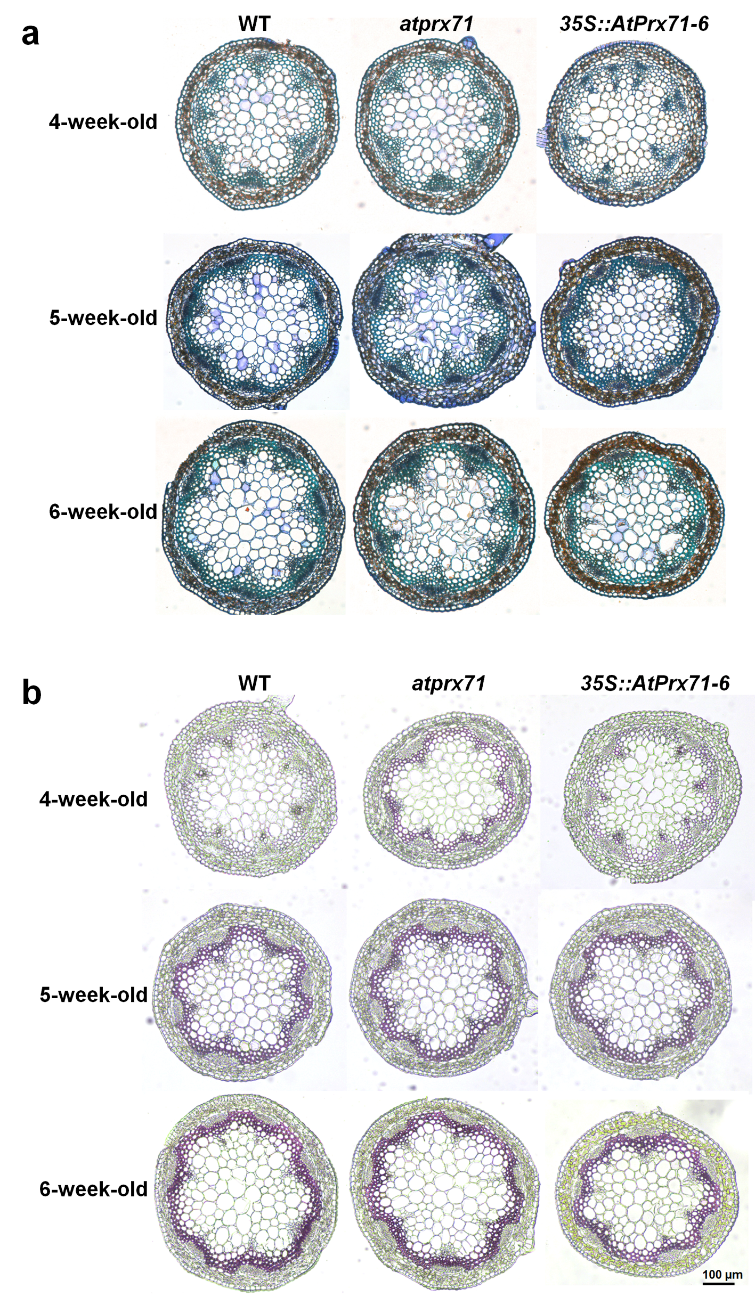


**Supplementary Figure S4** Histochemical analysis of stem sections from 4-week-old wild-type, *atprx71*, and *35S::AtPrx71-6* plants. Microscopic observations of the cross sections stained with toluidine blue (**a**) and phloroglucinol-HCl (**b**). Bar = 100 µm.

**Supplementary Table S1**

The top three peroxidases showing a high degree of amino acid identity to AtPrx71 and CWPO-C in selected plant species
